# Theory Driven Adaptation of an Elder Abuse Consultation Service

**DOI:** 10.1093/geroni/igaf122.087

**Published:** 2025-12-31

**Authors:** Elizabeth Bloemen, Sarah Tietz, Josh Bollan, Sarah Cox, Daniel Lindberg

**Affiliations:** University of Colorado, Aurora, Colorado, United States; University of Colorado School of Medicine, Aurora, Colorado, United States; University of Colorado School of Medicine, Aurora, Colorado, United States; University of Colorado Hospital, Aurora, Colorado, United States; University of Colorado School of Medicine, Aurora, Colorado, United States

## Abstract

Elder abuse consultation services are a new model of care for victims of elder mistreatment who are seen in the emergency room or hospital setting. Our team, the Vulnerable Elder Services, Protection, and Advocacy Team (VESPA) at the University of Colorado was developed and implemented in 2021. Consisting of specialized social workers and geriatricians, our team provides trauma informed care focused on reducing harm for an entire family system experiencing violence. In the first year, our team saw 42 cases and has grown to over 200 cases a year, having served over 700 victims and families to date. Due to changes in clinical volume; the improved recognition of abuse; and the challenges of the COVID 19 pandemic, our team has undergone multiple adaptations to best serve the victims and providers we care for. Our theory of change is that medical providers play a critical role in the response to elder mistreatment but that they are dis-incentivized from doing so by time constraints and a lack of training. Medical consultation teams offload this work, and the accompanying moral distress, thereby improve the recognition of abuse. As our team grew, and recognition grew, our consult numbers outpaced our funding. We have made several adaptations to our workflow to accommodate this while maintaining our core theory of change. These have included: limiting our response to stranger financial abuse; focusing on inpatient versus emergency department consultations; and developing a stepped model of care.

